# Development and validation of an intrinsic capacity composite score in the Longitudinal Aging Study Amsterdam: a formative approach

**DOI:** 10.1007/s40520-023-02366-2

**Published:** 2023-02-23

**Authors:** Kaisa Koivunen, Emiel O. Hoogendijk, Laura A. Schaap, Martijn Huisman, Martijn W. Heymans, Natasja M. van Schoor

**Affiliations:** 1 Epidemiology and Data Science, Amsterdam UMC Location Vrije Universiteit Amsterdam, De Boelelaan 1117, Amsterdam, The Netherlands; 2 Faculty of Sport and Health Sciences and Gerontology Research Center, University of Jyväskylä, Rautpohjankatu 8, P.O. Box 35, 40014 Jyväskylä, Finland; 3 Department of General Practice, Amsterdam UMC Location Vrije Universiteit Amsterdam, De Boelelaan 1117, Amsterdam, The Netherlands; 4 Aging and Later Life, Amsterdam Public Health Research Institute, De Boelelaan 1117, Amsterdam, The Netherlands; 5 Department of Health Sciences, Faculty of Science, Amsterdam Public Health Research Institute, Amsterdam Movement Sciences, Vrije Universiteit Amsterdam, De Boelelaan 1081, 1081 HV Amsterdam, The Netherlands; 6 Department of Sociology, Vrije Universiteit Amsterdam, De Boelelaan 1105, 1081 HV Amsterdam, The Netherlands

**Keywords:** Functional ability, Healthy aging, Measurement, WHO

## Abstract

**Background:**

Intrinsic capacity (IC) defined by the WHO refers to the composite of five domains of capacities. So far, developing and validating a standardized overall score of the concept have been challenging partly because its conceptual framework has been unclear. We consider that a person’s IC is determined by its domain-specific indicators suggesting a formative measurement model.

**Aims:**

To develop an IC score applying a formative approach and assess its validity.

**Methods:**

The study sample (*n* = 1908) consisted of 57–88-year-old participants from the Longitudinal Aging Study Amsterdam (LASA). We used logistic regression models to select the indicators to the IC score with 6-year functional decline as an outcome. An IC score (range 0–100) was constructed for each participant. We examined the known-groups’ validity of the IC score by comparing groups based on age and number of chronic diseases. The criterion validity of the IC score was assessed with 6-year functional decline and 10-year mortality as outcomes.

**Results:**

The constructed IC score included seven indicators covering all five domains of the construct. The mean IC score was 66.7 (SD 10.3). The scores were higher among younger participants and those who had lower number of chronic diseases. After adjustment for sociodemographic indicators, chronic diseases, and BMI, a one-point higher IC score was associated with a 7% decreased risk for 6-year functional decline and a 2% decreased risk for 10-year mortality.

**Conclusions:**

The developed IC score demonstrated discriminative ability according to age and health status and is associated with subsequent functional decline and mortality.

**Supplementary Information:**

The online version contains supplementary material available at 10.1007/s40520-023-02366-2.

## Introduction

In 2015, the World Health Organization (WHO) introduced a new model for healthy aging, which focuses on trajectories of functional ability during different life phases [1]. According to this model, functional ability is determined by the continuous interaction between the intrinsic capacity (IC) of an individual and the relevant environmental characteristics. IC is defined as *a composite of all the physical and mental attributes on which an individual can draw upon during his/her life* [2]. Optimizing the trajectories of IC as well as enhancing adaptation to losses in IC through environmental facilitators and compensation strategies help to maintain functional ability and foster healthy aging.

The IC construct was developed based on the International Classification of Functioning, Disability and Health (ICF) framework and prior empirical evidence on factors known to be important risk factors of functional loss during aging [2]. Five key domains, namely, locomotion, cognition, sensory, psychology, and vitality, were proposed to be key components of the IC construct [2]. Although several validated measurements already exist to capture most of the IC domains separately, there has been a need for monitoring individual’s overall capacity with a composite score [3]. A score summarizing complex and multi-dimensional constructs is often easier to use and interpret than a profile of many separate indicators [4]. However, operationalizing complex constructs, such as IC, into one score is not straightforward and methodological aspects to consider are vast.

**Fig. 1:**
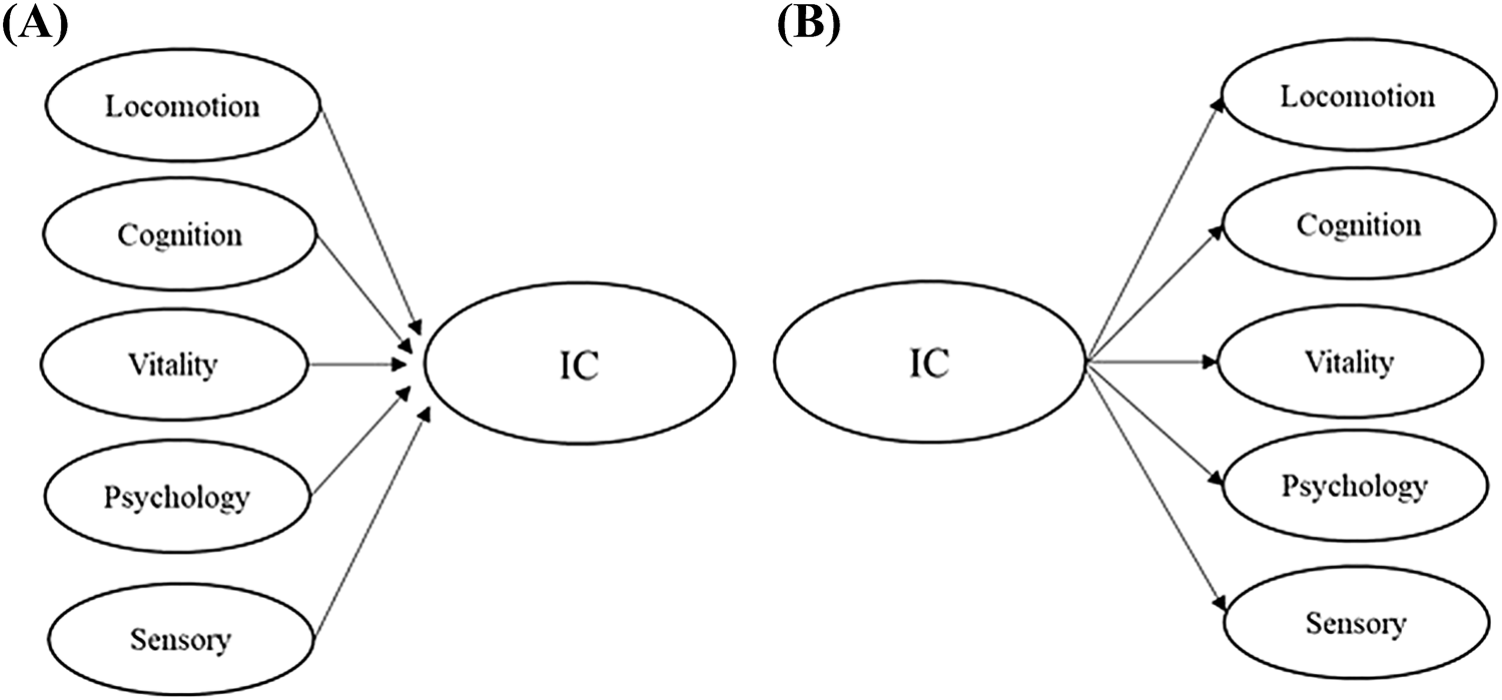
**A** A formative measurement model: The overall intrinsic capacity (IC) represents the composite or aggregate of capacities of different domains and levels of functioning. **B** A reflective measurement model: IC is a general underlying trait, which causes the variation in the observed indicators across different domains of functioning

Currently, IC measurements have been constructed using data from prospective cohort studies on aging but translating the new concept into a standardized composite measurement has proved to be challenging partly because the conceptual and measurement model of IC has not been clear. In a recent scoping review of our group, we concluded that IC should be considered as a formative construct [5]. This means that the person’s overall IC is determined by the domain-specific indicators, and therefore, it can be measured as an aggregate of capacities (Fig. 1). In other words, a person’s overall IC is determined by his or her capacities with regard to locomotion, cognition, sensory, psychology, and vitality, and not vice versa. However, up till now, most studies developing and validating IC scores have applied a reflective approach, in which IC is considered as an underlying general factor causing the changes in the observed capacities across all the five domains [6–8]. However, although the IC domains are interrelated and represent capacities, which require functioning of several body systems (e.g., organs and tissues), they may not share the same physiological basis or follow similar trajectories during aging. Thus, a general underlying capacity (Fig. 1) factor may not be an accurate assumption to base the IC construct and score on.

In formative measurements, the indicators in the score are expected to explain different aspects of the construct and not common variance [9]. However, for formative models, measurement theories are less developed compared to the reflective model, and there are no clear guidelines for the selection of indicators [9, 10]. From a theoretical perspective, the selected indicators in the score should cover the entire scope of the construct, whereas from a practical perspective, it is desirable to create a summary score that is informative without having excessive numbers of indicators [9]. So far, the most recommended method for selecting the indicators has been based on how well they relate to external measures that summarize the essence of the construct or are theoretical outcomes of it [11].

In the present study, we aimed to develop and validate an IC score using data from the Longitudinal Aging Study Amsterdam (LASA) by applying a formative approach. We used multiple regression to identify and select the most significant indicators to the IC score using 6-year functional decline as an outcome, which, according to the WHO’s healthy aging model, can be assumed to be an outcome of declined IC [1]. In addition, we assessed the structural validity of the score by evaluating whether the selected indicators represent all the five domains of the construct as well as known groups and criterion validity of the constructed summary score.

## Methods

### Design and study sample

We utilized data from the Longitudinal Aging Study Amsterdam (LASA), which is an ongoing longitudinal study consisting of a nationally representative sample of the Dutch older population [12]. Briefly, a random sample was drawn from population registers from 11 municipalities in the Netherlands. The LASA study started in 1992/1993 consisting of 3107 participants aged 55–85 years. Since then, data are collected approximately every 3 years with a face-to-face main interview and a medical interview, which also includes performance tests in the homes of the respondents. In 2002/2003, a second and in 2012/2013, a third refresher cohort of participants aged 55–64 years were added using the same sampling frame as the original cohort. The study was approved by the Ethical Review Board of the VU University Medical Center. All participants signed an informed consent before participating in the study.

For the current study, data from main and medical interviews of the first two LASA cohorts were combined, with baseline measurements in 1995/1996 (aged ≥ 65 years) and in 2005/2006 (aged ≥ 57 years). These measurement cycles were used for the baseline analyses, since not all relevant variables for operationalizing IC were available at the first LASA measurement cycles of the cohorts in 1992/1993 and 2002/2003, respectively. Follow-up outcome data on functional limitations were drawn from the measurement cycles conducted in 2001/2002 and 2011/2012, for the first and second cohort, respectively.

**Fig. 2:**
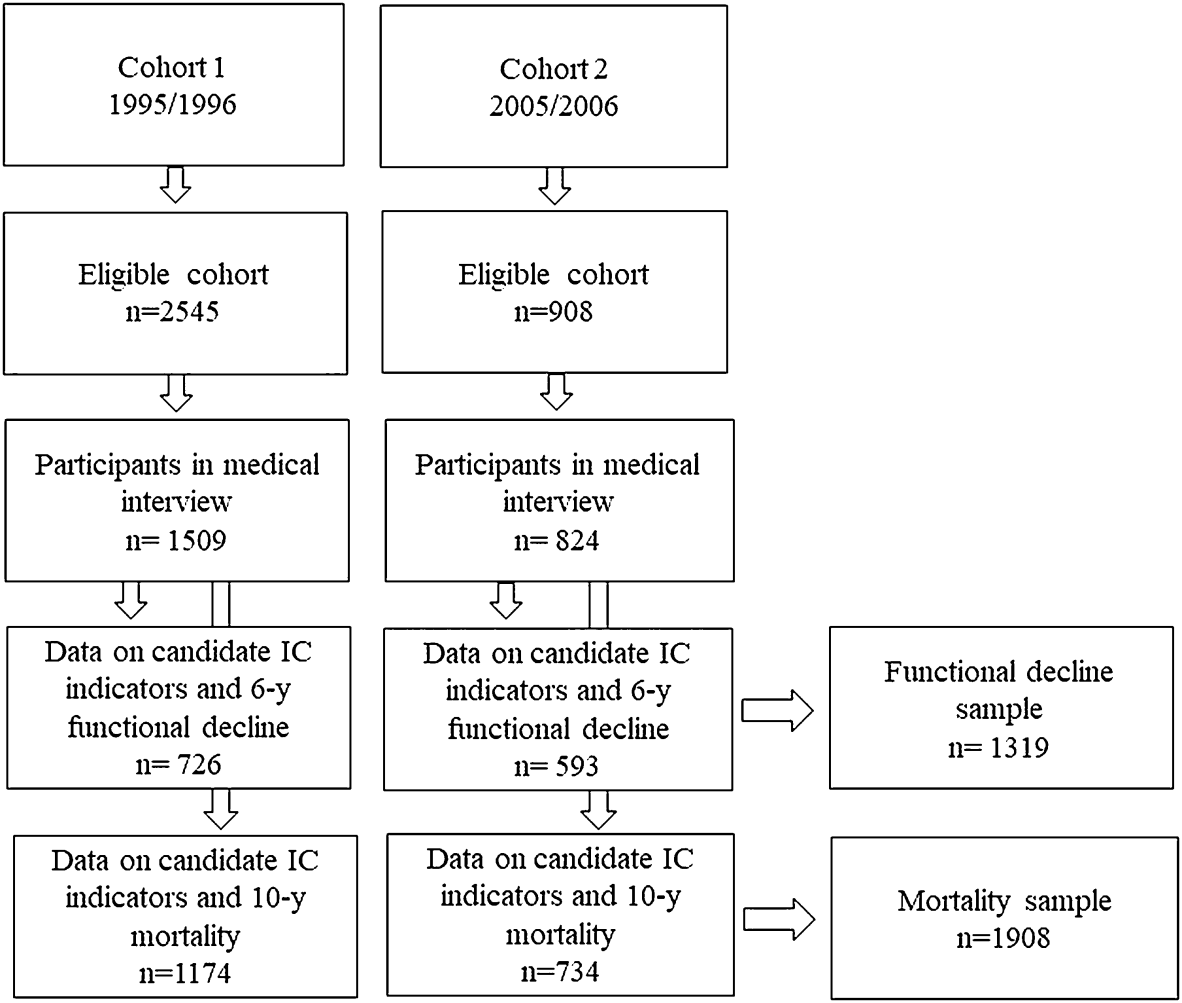
Flowchart of the study population. Note: In cohort 1, only persons aged 65 and over were selected for the medical interview, and therefore, younger participants from this cohort were not included in the current analyses

The study sample consisted of respondents who participated in both main and medical interviews, who had data on all candidate IC indicators at baseline, and who had data on at least one of the outcome measurements, that were 6-year functional decline and 10-year mortality. Mortality status was available for all participants, but due to missing data on baseline variables, in total, 1908 participants were eligible in the analyses on mortality. During the 6-year follow-up, 552 participants dropped out (due to, e.g., mortality or refusal), and 37 participants did not have complete data on functional limitations at follow-up. As a result, 1319 participants were included in the analytical sample using functional decline as an outcome (Fig. 2).

### Measurement of IC

To ensure the content validity of the score, we considered measurements of indicators collected in LASA that fit best to the conceptualization of the IC construct. Selection of potential indicators was guided by the following criteria: the indicator (1) has been identified as a predictor of health and functional decline during aging in prior literature, (2) has preferably continuous scoring and is able to detect low and high capacities in one of the five defined key domains of IC, (3) can be easily administered and incorporated in routine clinical practice, and (4) is available at different LASA measurement waves, to have the opportunity to study changes in LASA-IC score over time in future research. The following indicators measured at baseline were considered to cover the five domains:

*Vitality* was measured with hand grip strength [13], which was assessed with a strain-gauged dynamometer (Takei TKK 5001; Takei Scientific Instruments Co. Ltd., Tokyo, Japan). Participants performed two maximum forced trials with both hands in a standing position with the arm along the body. The total hand grip score was calculated summing and dividing by two the maximum values of right and left hands.

*Locomotion* was assessed with three indicators: walking speed, chair rise test, and standing balance test. Walking speed was measured as time (seconds) needed to walk 3 m, turn around, and then walk back 3 m as fast as possible. In the chair rise test, participants fold their arms across the chest and the time to perform five sit-to-stand repetitions was measured in seconds. Standing balance was measured with feet in the tandem position for a maximum of 10 s.

*Cognition* was assessed with three tests covering memory, information processing speed, and general cognitive functioning. Memory was measured with a 15 Words Test (15WT), which is a Dutch version of the Auditory Verbal Learning Test [14, 15]. In the test, the participant is instructed to learn 15 one-syllable nouns, which are read aloud by the interviewer. The same word list is repeated in three trials, and after each, the participant is asked to recall as many words as possible. The maximum number of correctly remembered words was used in the analyses, which measures immediate memory.

Information processing speed was assessed by a Coding task, which is an adjusted version of the Alphabet Coding Task, which is a letter substitution task [16, 17]. In the assessment, two rows of characters were shown; each character in the upper row belongs to a character in the lower row. In the test itself, one row contains characters and the other is empty. The participant is instructed to complete as many two character combinations as possible by naming the corresponding character. The total assessment consists of three trials of 1 min and the score of each trial is defined as the number of completed combinations irrespective of the number of wrong answers [18]. The score of these three trials was used in the analyses.

General cognitive functioning was measured with the Mini-Mental State Examination (MMSE), which is a test consisting of 23 items representing seven domains of cognitive functioning: orientation in time, orientation in place, registration of three words, attention and calculation, recall of three words, language, and visual construction [19]. The score ranges from 0 to 30 with a higher score indicating better cognitive capacity.

The *Psychology* domain consisted of measures of depressive symptoms, anxiety, mastery, and self-efficacy. Depressive symptoms were assessed with the Center for Epidemiologic Studies Depression Scale (CES-D) scale [20]. The scale consists of 20 items measuring depressive symptoms experienced in the past week. The total score ranges from 0 to 60, with higher scores indicating more severe symptoms. Anxiety was measured with the anxiety subscale of the Hospital Anxiety Depression Scale (HADS-A) consisting of seven items [21]. In the adaptation to LASA, the response options range from 1 (rarely or never) to 4 (mostly or always) and the sum score ranges from 0 to 21 with higher scores indicating higher anxiety. In the score construction, imputation was performed for participants who had one missing item by calculating the average of the six available items.

Mastery refers to a sense of being in control of events and ongoing situations and was measured with the Pearlin Mastery Scale [22], which consists of five items. The total score ranges from 5 to 25 with higher scores indicating higher sense of mastery. Self-efficacy, which is the belief of a person of in own ability to organize and execute certain behaviors, was measured with a 12-item version of the General Self-Efficacy Scale (GSES-12), which total score ranges from 12 to 60 with higher scores indicating higher self-efficacy [23].

The *Sensory* domain was assessed with self-rated items of vision and hearing. Vision was assessed with three items: “*Can you read the normal, small print in the newspaper without glasses or contact lenses?”,* “*Can you recognize someone’s face from a distance of 4 m without glasses or contact lenses?*”, and *“Can you see well enough?”*. Hearing was assessed also with three items: “*Can you follow a conversation in a group of three or four persons without an aid?*”, “*Can you follow a conversation with one person without an aid?*”, and “*Can you hear well enough?*”. The response options in each item ranged from one to four: 1 (Yes, without difficulty), 2 (Yes, but with some difficulty), 3 (Yes, but with much difficulty), and 4 (No, I cannot).

### Outcomes

According to the WHO’s healthy aging model, the level of IC largely defines the individual’s functional ability in interaction with the surrounding environment [24]. In line with this, functional decline can be assumed to be a consequence of declined IC and we used it as an outcome to select the indicators to the IC summary score. As one of the motives of monitoring IC score is to evaluate the risk of individual’s future functioning and adverse outcomes to target preventive interventions [6], we examined whether the constructed full summary score is associated with subsequent functional decline and mortality.

*Functional decline* was assessed with six items of functional limitations at baseline and 6-year follow-up*.* The participants were asked to evaluate the degree to which he/she had difficulty performing six usual daily activities: standing up and sitting down on a chair, cutting own toenails, walk outside during 5 min without stopping, walking up and down a staircase of 15 steps without resting, using own or public transportation, and dressing and undressing oneself. The response options ranged from one to five: 1 (yes, without help), 2 (yes, with some difficulty), 3 (yes, with much difficulty), 4 (only with help), and 5 (no, I cannot). The responses were summed to a total score, which ranged from 0 to 30 with higher scores indicating more functional limitations. Functional decline was defined with the Edwards-Nunnally (EN) index, which captures significant change in the functional limitations score by taking into account measurement error and regression to the mean [25]. The following formula was used: XT2 > (Cronbach’s α × (XT1 − mean) + mean − 1.96 × standard error). XT1 and XT2 refer to the individual’s raw score on T1 and T2, respectively.

Information on *mortality* (vital status and date of death) was retrieved from the registers of the municipalities in which the respondents were living. We determined 10-year mortality since the date of the main interview.

### Other variables

Background characteristics included age at baseline, sex, the number of self-reported chronic diseases, body mass index (BMI), and educational level. The number of chronic diseases ranged from 0 to 9 and was calculated based on the most frequently occurring somatic chronic disease in the Netherlands, that are, chronic non-specific lung disease, cardiac disease, peripheral artery diseases, diabetes mellitus, cerebrovascular accident or stroke, osteoarthritis, rheumatoid arthritis and/or cancer, and a maximum of two other chronic diseases which symptoms lasted for at least 3 months. For analyses, the variable was categorized into four categories: none, one, two, and three or more chronic conditions. Participants’ height and weight were measured using a stadiometer and a calibrated bathroom scale. BMI was calculated by dividing the body weight (in kg) by the square of the body height (in m). Education was categorized into low (elementary education or less), middle (lower vocational education and general intermediate education), and high education (intermediate vocational education, general secondary education, higher vocational education, college education, and university) [26].

### Statistical analyses

#### Indicator selection

First, after stratification by sex, we rescaled all the candidate indicators of IC using the percent of maximum possible (“POMP”) method [27–29], so that the variables had the same unit. After rescaling, all variables ranged from 0 (low capacity) to 100 (high capacity).

We used logistic regression models to select the indicators to the IC score with 6-year functional decline (yes/no) as an outcome. Before fitting multivariable logistic regression models, we assessed collinearity among the candidate indicators with Pearson correlation. We considered two indicators as multicollinear with a correlation of ≥ 0.8 [30]. In case of multicollinearity, the indicator with the strongest association with functional decline was selected to the model. The highest correlation was detected between CES-D and anxiety (*r* = 0.71) and no candidate indicators were excluded.

In the multivariable logistic regression models, we applied a stepwise backward elimination procedure to exclude indicators that were not statistically significant (*p* > 0.05). In case of all indicators of one of the five IC domains were excluded from the model, we selected the last indicator omitted from the domain in question to cover the full IC construct. To test stability of the indicator selection and combination of selected indicators, we applied bootstrapping techniques using 2000 samples [31] and calculated the frequency of indicator and model selection. The indicator was included in the IC score if it was selected in ≥ 50% of the samples.

#### IC score construction and validation

The IC score was constructed of the indicators included in the final regression model. We calculated first a mean score for domains with multiple indicators before calculating a mean score over all five domains. We tested known-groups’ validity for construct validity [32] of the IC score based on the hypotheses that the IC is lower among older age groups and people with poorer health. Known-groups’ validity was tested by comparing the IC scores between five age groups and four categories of chronic diseases with one-way ANOVA and Tukey’s HSD test for multiple comparisons.

To assess criterion validity of the constructed IC score, we analyzed bi- and multivariable logistic regression and Cox proportional hazard models to study the associations of the IC score with 6-year functional decline and ten-year all-cause mortality, respectively. The multivariable models were adjusted for age, sex, birth cohort, number of chronic diseases, and BMI.

For the analyses, we used IBM SPSS Statistics 28 (SPSS Inc., Chicago, IL) and R for Windows version 4.2.1 (R Development Core Team, Vienna, Austria: R Foundation for Statistical Computing), and the statistical significance was set at *p* < 0.05.

## Results

Table 1 shows the characteristics of the two analytical samples. In total, 184 participants (14%) experienced functional decline during 6-year follow-up of the sample used for the analyses with functional decline as an outcome. Of the participants in the sample used in mortality analyses, 553 (29%) deceased within 10 years after baseline. The functional decline sample consisted of participants who were slightly younger and reported less functional limitations at baseline compared to the mortality sample. The participant characteristics in the two included birth cohorts included in the analyses are presented and compared in the Supplementary Table 1. The second cohort with baseline measurements in 2005/2006 was younger than the first cohort with baseline measurements in 1995/1996. In addition, the participants in the second cohort had higher education, less chronic diseases and functional limitations, and higher BMI at baseline than the participants in the first cohort. During the follow-up, a smaller proportion of the participants in the second cohort experienced functional decline or deceased.

**Table 1:** Characteristics of the analytical samples

Variable	Functional decline sample (*n* = 1319)	Mortality sample (*n* = 1908)
Age (years)	68.6 ± 7.0	70.3 ± 7.8
Sex (female)	690 (52)	954 (50)
Education		
Low	371 (28)	595 (31)
Middle	463 (35)	651 (34)
High	484 (37)	661 (35)
Number of chronic diseases		
None	273 (21)	376 (20)
One	464 (35)	613 (32)
Two	326 (25)	497 (26)
Three or more	256 (19)	422 (22)
BMI (kg/m^2^)	27.2 (4.1)	27.1 (4.2)
Functional limitations at baseline (0–30)	7.3 ± 2.5	7.7 ± 3.0
Functional decline at 6-year follow-up	184 (14)	–
Deceased after 10-year follow-up	–	553 (29.0)

Data are presented as mean ± SD or *n* (%)

*BMI* Body Mass Index

In the final model of the stepwise backward logistic regression (Table 2), 7 of the 17 candidate indicators of IC were significantly associated with functional decline at follow-up. All the five domains of the IC construct were covered, and no domains had to be forced into the model. Vitality, cognition, and psychology domains each were represented by a single indicator (grip strength, coding, and self-efficacy, respectively). The locomotion domain was represented by two indicators (walking speed and balance) and the sensory domain was covered by single items of vision and hearing. Bootstrapping showed stability of the indicator selection as all the indicators included in the final model were selected over 50% of the 2000 samples. The selection frequency ranged from 70.8% (hearing: following conversation in a group) to 98.3% (grip strength). In addition, the final model was selected most of the times (5% of the samples) among several other competing models, meaning that the combination of the indicators in the final model was the most stable when compared to other possible combinations. The selection frequencies of all the candidate indicators and the ten most stable models are presented in Supplementary Table 2.

**Table 2:** The final logistic regression model with functional decline as an outcome developed in combined data of 58–88-year-old people from the two LASA cohorts (*n* = 1319)

Domain	Indicator	OR (95% CI)	*p* value	Selection frequency (%)^a^
Vitality	Grip strength	0.97 (0.96–0.98)	< 0.001	98.3
Locomotion	Walking speed	0.94 (0.90–0.99)	0.010	82.4
	Balance	0.99 (0.99–1.00)	0.002	82.0
Cognition	Coding	0.98 (0.97–0.99)	< 0.001	87.8
Sensory	Vision: distance	0.99 (0.99–1.00)	0.001	81.8
	Hearing: following conversation in a group	0.99 (0.99–1.00)	0.005	70.8
Psychology	Self-efficacy	0.98 (0.97–0.99)	0.001	74.1

*OR* odds ratio per 1 point, *CI* confidence interval

^a^The selection frequencies of the IC candidate indicators as a result of the bootstrap selection procedures

### Intrinsic capacity (IC) score

**Fig. 3:**
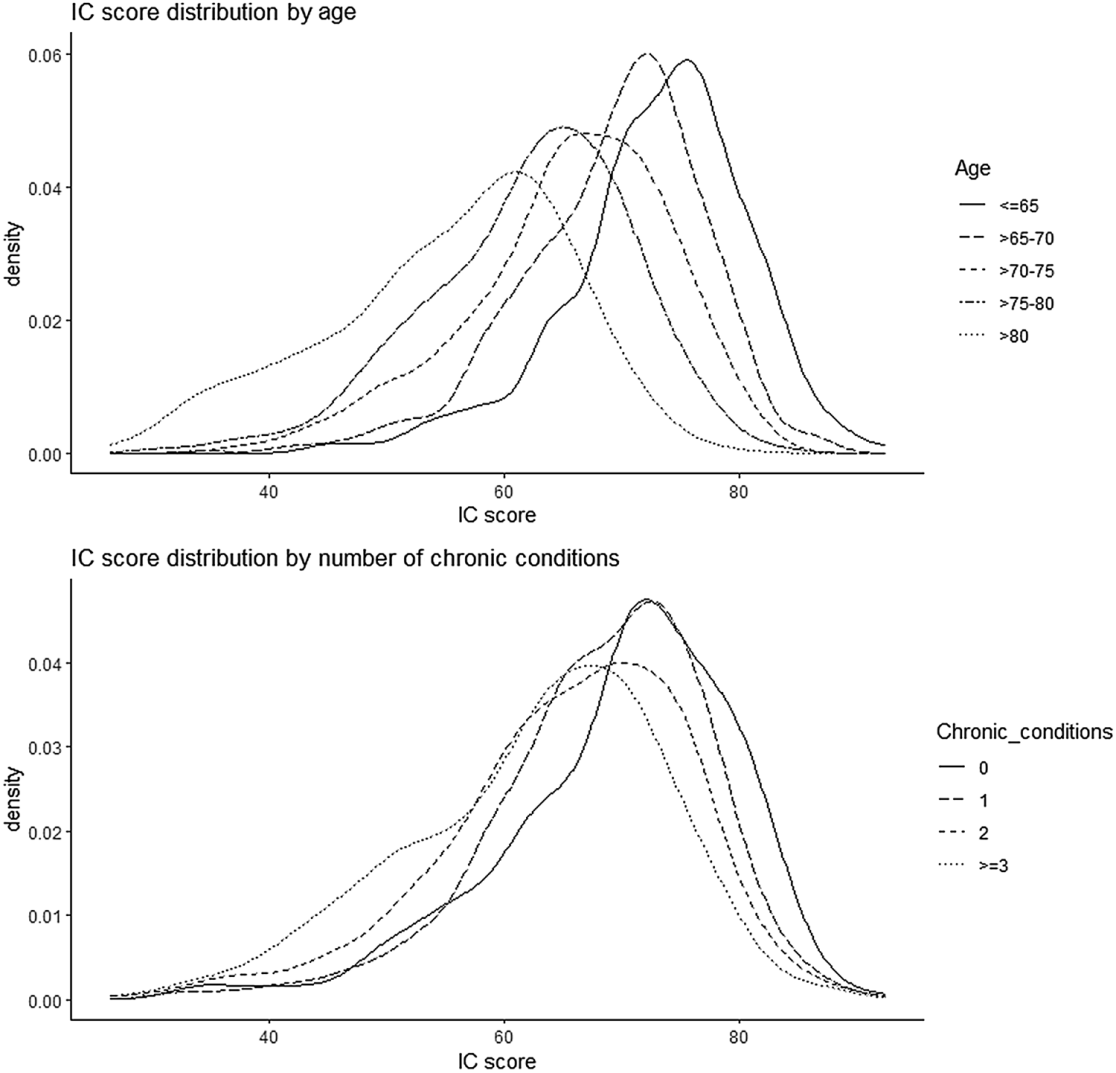
Distribution of the IC score at baseline by age and number of chronic diseases categories (*n* = 1908)

A mean score was calculated first for domains with multiple indicators (locomotion and sensory), after which the full IC score was calculated over the five domains ranging from 0 to 100. Analyzed with the larger analytical sample (*n* = 1908), the mean IC score was 66.7 (SD 10.3). Figure 3 shows the distribution of the IC score at baseline by age and number of chronic diseases categories. The distribution of IC values was skewed to the left, especially in younger age groups, showing that they had higher levels of IC and less variation in the scores. A one-way ANOVA showed that the differences were statistically significant (*f*(4) = 237.6, *p* < 0.001). In the multiple group comparisons (Supplementary Table 3), only the age groups of > 70–75 and > 75–80 years did not differ statistically in their mean IC scores. In addition, participants with lower number of chronic diseases had higher IC scores (*f*(3) = 41.08, *p* < 0.001). In the group comparisons, only those who reported none or one chronic disease did not differ statistically in their IC level.

The results from the logistic regression models showed that a one-point lower IC score was associated with a 10% increase in the probability for functional decline over 6-year follow-up (OR 0.90; 95% CI 0.89–0.92; Table 3). Adjustment for age, sex, and birth cohort attenuated the association slightly (OR 0.93; 95% CI 0.91–0.95). In addition, a one-point higher IC score was associated with a 6% decreased mortality hazard in the unadjusted model (HR 0.94; 95% CI 0.93–0.95). The association remained after adjustment for age, sex, and birth cohort (HR 0.98; 95% CI 0.97–0.99).

**Table 3:** Associations of the IC score with functional decline and mortality

Outcomes	6-Year functional decline		10-Year mortality	
	Model 1	Model 2	Model 1	Model 2
	OR (95% CI)	OR (95% CI)	HR (95% CI)	HR (95% CI)
IC score (per 1 point)	0.90 (0.89–0.92)	0.93 (0.91–0.95)	0.94 (0.93–0.95)	0.98 (0.97–0.99)
Age (per 1 year)		1.04 (1.09–2.25)		1.09 (1.07–1.10)
Sex (women vs. men)		1.56 (1.01–2.25)		0.54 (0.45–0.65)
Birth cohort (2 vs. 1)		0.37 (0.20–0.66)		0.64 (0.46–0.88)
Number of chronic diseases				
None		Ref		Ref
One		2.13 (1.16–4.16)		0.97 (0.74–1.28)
Two		2.03 (1.07–4.03)		1.31 (0.99–1.72)
Three or more		2.53 (1.33–5.05)		1.76 (1.34–2.31)
BMI, per 1 kg/m^2^		1.09 (1.05–1.13)		0.98 (0.96–1.00)

*OR* odds ratio, *HR* hazard ratio, *CI* confidence interval

## Discussion

IC is suggested to be a comprehensive measure of individuals’ reserve capacities and health status that tend to decline with aging [24]. In the current study, we developed an IC summary score in LASA among participants aged 57–88 years by applying for the first time a formative measurement approach. The developed score consisted of seven indicators covering all five domains of the IC construct. The score showed to be higher in younger age groups and people with fewer chronic diseases, which indicates that the score is able to detect age-related differences in functional capacities and that it is also able to discriminate individuals according to health status. The score also demonstrated a strong association with respect to subsequent functional decline and mortality, and thus, it may provide useful information with regard to a person’s future functional ability and health.

Indicators covering all the five domains of IC were selected in the score in the final logistic regression model and there was no need to force any indicators into the score only for conceptual reasons. This suggests that the selected indicators did not overlap substantially but represented unique and meaningful dimensions of the IC construct consisting of five domains. The findings of the indicator selection procedure may also inform researchers about possible weights for different indicators/domains for future studies. Our results showed that walking speed was most strongly associated with the risk of developing functional decline, which suggests higher relative importance of the locomotion domain.

Our study is the first attempt to develop and validate an IC score with a formative measurement approach. We have previously argued that the IC construct is defined by its five domains (or indicators in these domains) and should be measured as an aggregate of these different dimensions of capacities [5]. Thus, the conventional psychometric methods relying on the reflective model, such as factor analysis and estimates of internal consistency, were not suitable to select indicators and evaluate structural validity of the score. The formative approach is seldom recognized in health and medical research, although many multi-dimensional measurements in this field could be operationalized with composite indices rather than reflective scales. In this article, we demonstrate one way to conduct summary score development with formative constructs using multiple regression. This method aligns with the other approaches suggested to handle formative models, but which have applied structural equation modelling with outcome indicator(s) [9, 11]. In our analysis, we selected the IC indicators and assessed the structure of the constructed IC score using subsequent functional decline as an outcome variable. It is important to note that the aim was not to define an optimal prediction model of functional decline, which may also include many other factors [33], but to select the indicators within the IC concept. The choice of the outcome was based on the theoretical assumption of the Healthy Ageing framework, which states that declined IC constitutes a risk to develop functional limitations [1]. An unavoidable drawback of this approach is that the indicator elimination and selection procedures rely strongly on a single measurement of functioning. However, the subsequent analyses showed that the developed IC score functions in expected ways also in relation to other variables (i.e., age, chronic diseases, and mortality). Nevertheless, validation of an instrument is an ongoing process and applicability of the developed score still needs to be tested in other settings, for example, to investigate whether it is sensitive to detect changes longitudinally and in relation to other outcomes related to healthy aging, such as quality of life and social participation.

In the current study, we used similar indicators to measure IC as in the previous studies using large cohort studies of older adults [6, 7, 29]. Due to our indicator selection procedure and the intention to exclude indicators having little additional value for the summary score, the developed IC score consisted of a smaller number of variables as the operationalization of IC by Beard et al. [6, 7]. When compared to the IC measure used by Stolz et al. [29], which consisted of eight indicators, the IC measure developed in the current study showed similar associations with respect to functional decline and mortality. Contrary to other studies, indicators of positive psychological capacities (mastery, self-efficacy) were available in our data in addition to measurements related to emotional distress (depression, anxiety). Interestingly, the measure of self-efficacy was included to the summary score over depression, which is mainly used in other developed IC measurements [34]. This may be explained by the fact that depressive symptoms have been shown to be constantly associated with many other capacities, such as muscular strength [35], physical disability [36, 37], and cognitive deficits [38]. Therefore, depressive symptoms may have overlapping variance with other indicators, and aspects of positive psychological capacities can make the summary score more multifaceted and holistic. Nevertheless, in future studies, it may be useful to compare IC measurements based on different development procedures as well as a replication of the approach used in this study.

Aggregating multiple indicators into one score leads to the loss of information about the different contributing aspects, although for practical reasons, it is often useful [10]. The IC summary score may provide an important measure of an individual’s level of overall functional capacity [39] and it may serve as a tool for researchers and policymakers to monitor and compare IC among different populations. Considering the individual’s overall IC as a sum of its parts fits also well to the clinical applications of IC as the overall capacity can be improved by targeting its constituent domains or indicators. The summary score may provide a general view of patients’ health in clinical work although it is still important to consider more specific information at a person level [6, 40] when it also may be useful to include additional capacity indicators that were not included in the developed IC summary score. In addition, it is noteworthy that, although treated as separate entities, the capacity domains or indicators are not isolated but interact with each other as part of a system [2], which may make it feasible to examine the relationships among indicators to gain more insight in the dynamics of IC.

The strengths of our study include consideration of the conceptual framework and measurement model of IC that was used as the foundation for the score development, a large nationally representative sample of older adults in the Netherlands with longitudinal study design and mainly performance-based and continuous measurements from different domains of functioning. In addition, the bootstrapping techniques enabled us to examine the robustness of selection of individual indicators and the combination of the selected indicators, which strengthens the internal validity of the created IC score. There are also some limitations to our study. The lack of complete data in the candidate indicators of IC at baseline and attrition of participants during the follow-up restricted the study samples, which could mean that the participants represented a healthier section of the target population and selection bias may have occurred limiting the generalizability of the results. Selective drop-out may have led to an underestimation of functional decline during the follow-up. Future studies should further examine the external validity of the constructed IC score with larger and less-restrictive population-based samples but also in clinical populations. In the analyses, we combined to birth cohorts to increase sample size and to include younger participants, because according to the WHO’s healthy aging model, it is important to measure IC also in younger populations before the onset of functional limitations [1]. The birth cohort seemed to have a large impact on the outcomes in the regression analyses, which most likely is explained by the age differences between the cohorts. Therefore, in the future studies, it would be important to further explore birth cohort differences in IC and outcomes with comparable samples, which was beyond the scope of the current study. Finally, our data did not include performance-based measures of vision and hearing. Self-assessments may not provide as explicit and standardized information of sensory capacities as performance-based tests, and the restricted number of response options in the items limits the information about the full spectrum of capacity, especially from the end of higher functioning.

In conclusion, this study provides an approach to develop an IC summary score based on formative measurement model that is in line with the conceptualization of the multi-dimensional IC construct. The developed IC summary score demonstrated discriminative ability between age groups and according to health status and showed to be associated with subsequent functional decline and mortality.

## Supplementary Information

Below is the link to the electronic supplementary material.

Supplementary file1 (DOCX 23 KB)

## Data Availability

The datasets generated during the current study are not publicly available due to confidentiality, but the data underlying the results presented in this study are available from the Longitudinal Aging Study Amsterdam (LASA) and may be requested for research purposes. More information on data requests can be found on the LASA website: www.lasa-vu.nl.
