# Supplementary material for: Development and validation of an intrinsic capacity composite score in the Longitudinal Aging Study Amsterdam: a formative approach
Source: Aging Clin Exp Res. 2023 Feb 23;35(4):815–25. doi: 10.1007/s40520-023-02366-2 (PMC10115715; doi:10.1007/s40520-023-02366-2)
Supplement: Supplementary file 1 — Supplementary file1 (DOCX 23 KB) [file 40520_2023_2366_MOESM1_ESM.docx]

Supplementary table 1. Characteristics of the analytical samples in the two birth cohorts included in the analyses.

| **Variable** | **Functional decline sample (n=1319)** | | | **Mortality sample (n=1908)** | | |
| --- | --- | --- | --- | --- | --- | --- |
|  | Cohort 1 (n=726)  1995/1996 | Cohort 2 (n=593)  2005/2006 | p-value | Cohort 1 (n=1174) 1995/1996 | Cohort 2 (n=737) 2005/2006 | p-value |
| Age (years) | 73.2 ± 5.7 | 62.8 ± 3.0 | **<.001** | 74.8 ± 6.4 | 63.0 ± 3.0 | **<.001** |
| Sex (female) | 390 (54) | 300 (51) | .268 | 581 (50) | 373 (51) | .560 |
| Education |  |  |  |  |  |  |
| Low | 277 (38) | 94 (16) | **<.001** | 466 (40) | 129 (18) | **<.001** |
| Middle | 229 (32) | 234 (40) |  | 366 (31) | 285 (39) |  |
| High | 219 (30) | 265 (45) |  | 341 (29) | 320 (44) |  |
| Number of chronic diseases (self-reported) | 27.0 (4.0) | 27.5 (4.2) | **.024** | 26.9 (4.1) | 27.6 (4.3) | **<.001** |
| None | 130 (18) | 143 (24) | .053 | 209 (18) | 167 (23) | **.018** |
| One | 262 (36) | 202 (34) |  | 370 (32) | 243 (33) |  |
| Two | 187 (26) | 139 (23) |  | 319 (27) | 178 (24) |  |
| Three or more | 147 (20) | 109 (18) |  | 276 (24) | 146 (20) |  |
| BMI, (kg/m²) | 27.0 ± 4.0 | 27.5 ± 4.2 | **.025** | 26.9 ± 4.1 | 27.6 ± 4.3 | **<.001** |
| Functional limitations at baseline (0-30) | 7.7 ± 2.7 | 6.9 ± 2.1 | **<.001** | 8.2 ± 3.4 | 6.9±2.1 | **<.001** |
| Functional decline at six-year follow-up | 158 (22) | 26 (4) | **<.001** | -- | -- | -- |
| Deceased after ten-year follow-up | -- | -- | -- | 483 (41) | 70 (10) | **<.001** |

*Note.* Data are presented as mean ± SD or n (%).

Supplementary Table 2. Selection of variables as a result of the bootstrap model selection process

|  |  | Models selected (the first 10 are shown) | | | | | | | | | |
| --- | --- | --- | --- | --- | --- | --- | --- | --- | --- | --- | --- |
|  |  | 1 | 2 | 3 | 4 | 5 | 6 | 7 | 8 | 9 | 10 |
| Candidate indicator | Indicator selection (%) |  |  |  |  |  |  |  |  |  |  |
| Grip strength | 98.3 | x | x | x | x | x | x | x | x | x | x |
| Coding | 87.8 | x | x | x | x | x | x | x | x | x | x |
| Walking speed | 82.4 | x | x | x |  | x | x | x | x | x | x |
| Balance | 82.0 | x | x | x | x | x | x | x | x | x |  |
| Vision (distance) | 81.8 | x | x | x | x | x | x | x | x | x | x |
| Self-efficacy | 74.1 | x | x | x | x | x | x |  |  | x | x |
| Hearing (following conversation in group) | 70.8 | x | x | x | x |  |  | x | x |  | x |
| Chair stands | 34.3 |  | x |  |  |  |  | x |  | x | x |
| Vision (near) | 33.5 |  |  | x |  | x |  |  |  |  |  |
| Mastery | 30.3 |  |  |  |  |  |  |  |  |  |  |
| Seeing well enough | 14.8 |  |  |  |  |  |  |  |  |  |  |
| Hearing well enough | 11.1 |  |  |  |  |  |  |  |  |  |  |
| MMSE | 10.8 |  |  |  |  |  |  |  |  |  |  |
| Word test | 10.4 |  |  |  |  |  |  |  |  |  |  |
| Hearing (following conversation with one person) | 10.1 |  |  |  |  |  |  |  |  |  |  |
| CES-D | 8.8 |  |  |  |  |  |  |  |  |  |  |
| Anxiety | 5.4 |  |  |  |  |  |  |  |  |  |  |
| Model selection frequency (%) |  | 5 | 4 | 2 | 2 | 2 | 2 | 2 | 1 | 1 | 1 |

Supplementary Table 3. Comparison of the IC scores between the age groups and chronic diseases categories

| **Age (years)** | **IC score**  **(mean ± SD)** |  | **Number of chronic diseases** | **IC score**  **(mean ± SD)** |
| --- | --- | --- | --- | --- |
| ≤ 65 | 73.1 ± 7.75_a_ |  | None | 69.8 ± 9.93_a_ |
| > 65-70 | 69.3 ± 7.74_b_ |  | One | 68.3 ± 9.15_a_ |
| > 70-75 | 65.6 ± 8.58_c_ |  | Two | 65.5 ± 10.3_b_ |
| > 75-80 | 62.3 ± 8.66_c_ |  | Three or more | 62.8 ± 10.8_c_ |
| > 80 | 55.3 ± 10.2_d_ |  |  |  |

*Note.* Means sharing the same subscript are not significantly different from each other (Tukey's HSD, p<0.05)
